# Rapid evolution of increased vulnerability to an insecticide at the expansion front in a poleward‐moving damselfly

**DOI:** 10.1111/eva.12347

**Published:** 2016-01-27

**Authors:** Khuong Van Dinh, Lizanne Janssens, Lieven Therry, Hajnalka A. Gyulavári, Lieven Bervoets, Robby Stoks

**Affiliations:** ^1^ Institute of AquacultureNha Trang UniversityNha TrangVietnam; ^2^ Laboratory of Aquatic Ecology, Evolution and ConservationUniversity of LeuvenLeuvenBelgium; ^3^ Systemic, Physiological and Ecotoxicological Research GroupUniversity of AntwerpAntwerpBelgium

**Keywords:** agriculture, carryover effects, energy storage, evolutionary ecotoxicology, flight muscles, latitude, pyrethroids, range expansion

## Abstract

Many species are too slow to track their poleward‐moving climate niche under global warming. Pesticide exposure may contribute to this by reducing population growth and impairing flight ability. Moreover, edge populations at the moving range front may be more vulnerable to pesticides because of the rapid evolution of traits to enhance their rate of spread that shunt energy away from detoxification and repair. We exposed replicated edge and core populations of the poleward‐moving damselfly *Coenagrion scitulum* to the pesticide esfenvalerate at low and high densities. Exposure to esfenvalerate had strong negative effects on survival, growth rate, and development time in the larval stage and negatively affected flight‐related adult traits (mass at emergence, flight muscle mass, and fat content) across metamorphosis. Pesticide effects did not differ between edge and core populations, except that at the high concentration the pesticide‐induced mortality was 17% stronger in edge populations. Pesticide exposure may therefore slow down the range expansion by lowering population growth rates, especially because edge populations suffered a higher mortality, and by negatively affecting dispersal ability by impairing flight‐related traits. These results emphasize the need for direct conservation efforts toward leading‐edge populations for facilitating future range shifts under global warming.

## Introduction

Global warming is causing widespread poleward range expansions where species try to keep pace with their moving climate niche (Hickling et al. 2006; Chen et al. 2011). Under ongoing and more intense global warming, range‐expanding species are expected to continue to move more poleward to track their optimal thermal niche (Hickling et al. 2006; Chen et al. 2011). There is large variation in the rates at which different species’ geographic ranges expand in response to climate warming (Moritz and Agudo 2013; Mair et al. 2014), yet only part of the variation in these rates can be explained by species differences in intrinsic dispersal abilities (Angert et al. 2011; Fordham et al. 2013). Understanding factors shaping the speed of range expansion is timely as there is increasing concern that many species are too slow to track their moving climate niche (Razgour et al. 2013).

Pesticide exposure may be one notable factor that may affect range expansion as individuals have to cross‐agricultural landscapes with extensive use of pesticides. Moreover, the frequency of pesticide application is likely to increase under global warming, particularly at higher latitudes (Kattwinkel et al. 2011) where many edge populations are migrating to (Hickling et al. 2006). How species will deal with pesticides under global warming is becoming a major topic in ecotoxicology (Noyes et al. 2009; Moe et al. 2013), yet the expected interplay of range expansions and contaminants on organisms has been ignored. The vulnerability of edge populations at the moving range front to pesticides may slow down the range expansion in two ways. Firstly, pesticide exposure may impair the locomotory performance of animals by negatively affecting energy storage (e.g., Janssens et al. 2014) and muscles (e.g., Mehlhorn et al. 1999). Secondly, pesticides may reduce population growth rates by reducing larval growth rate and imposing mortality, thereby slowing down further range expansion.

During range expansions, edge populations may show rapid evolution as they experience novel evolutionary pressures because edge populations are assorted by dispersal ability and have a lower density of conspecifics than do core populations (Phillips et al. 2010). This rapid evolution entails a broad range of traits, including morphology, physiology, and behavior that are selected toward values that increase the rate of spread (Phillips 2009; Burton et al. 2010; Phillips et al. 2010; Shine et al. 2011; Brown et al. 2015). For example, edge populations at moving range fronts typically evolve a faster life history, a higher investment in reproduction (Phillips 2009; Phillips et al. 2010), higher activity levels (Therry et al. 2014b), an increased investment in locomotory ability (Hill et al. 2011) and a higher investment in immune function to avoid a reduction in dispersal rates (Therry et al. 2014c). Note that these evolutionary changes are not driven by adaptation to the range edge or any new biotic conditions met, but are driven by the dynamic process of range expansion itself. Therefore, these effects are only to be expected in edge populations at moving range fronts and not in edge populations at stable range fronts. These evolutionary changes require a higher allocation of energy toward growth and development and costly structures (such as muscles) and functions (such as immune function). Given that these investments in costly traits to accelerate range expansion will imply trade‐offs with other costly processes such as investment in detoxification and repair (Sibly and Calow 1989; Congdon et al. 2001), it is to be expected that edge populations at moving range fronts will be more vulnerable to stressors such as pesticides. This novel hypothesis needs explicit testing and would provide an extra dimension to the insight that ecotoxicology needs a macroecological (Beketov and Liess 2012; Clements et al. 2012) and evolutionary (Coutellec and Barata 2011; Hammond et al. 2012) perspective.

We tested for the potential role of pesticide exposure in slowing down range expansion and whether evolutionary processes during range expansion increase the vulnerability to pesticides. We studied this in a currently poleward‐moving damselfly by comparing replicated core and edge populations at low and high densities in an outdoor container experiment. Damselflies are among the taxa showing the strongest poleward range expansions (Hickling et al. 2006). They have a complex life cycle with an aquatic larval stage where growth occurs and a terrestrial flying adult stage where reproduction and dispersal occur (Stoks and Cordoba‐Aguilar 2012). We included a density treatment as densities are initially lower at the expansion front while pesticide effects may be apparent or stronger at high densities (e.g., Jones et al. 2011; Knillmann et al. 2012). We tested for effects on larval survival, growth, and development and for potential carryover effects bridging metamorphosis on a set of flight‐related traits (body mass, relative flight muscle mass, and fat content), that may be especially relevant for dispersal ability. As study species we chose the poleward range‐expanding damselfly *Coenagrion scitulum* (Swaegers et al. 2013). We have previously shown that this species evolved a faster life history (Therry et al. 2014b) and a higher investment in flight muscles and immune response (Therry et al. 2014c) at the range front. As pesticide we used esfenvalerate, a widely applied pyrethroid insecticide (Spurlock and Lee 2008; Stehle and Schulz 2015) that is highly toxic to aquatic invertebrates (Rasmussen et al. 2013), including damselfly larvae (Beketov 2004).

## Materials and methods

### Study populations and rearing experiment

*Coenagrion scitulum* is a Mediterranean damselfly preferring small ponds (Dijkstra 2006). Up to the 1990s the northern range limit was situated in northern France, after which a north‐eastward range expansion has occurred (Swaegers et al. 2013). In 2010, the northern‐most limit of the expanding range margin was situated in the southern parts of the Netherlands, and the northeastern limit in Western Germany. We studied two core populations and two edge populations. The two core populations, both in France, were situated in Nord‐Pas‐de‐Calais (+50°26′34.37″N, +1°35′08.81″E) and Indre (+46°43′14.03″N, +1°10′20.22″E). Both core populations are within the historical distribution of the species (Therry et al. 2014c). Note that the Nord‐Pas‐de‐Calais population is situated at the edge of the historical range as it is bordering the Atlantic Ocean, making our setup conservative as we only hypothesize a higher vulnerability to pesticides in edge populations at moving range fronts. The two edge populations were situated in Saarland (Germany, +49°14′52.96″N, +7°16′20.08″E) and in Zeeland (the Netherlands, +51°21′25.99″N, +3°40′01.37″E), both at the moving range front (further on we just call them ‘edge populations’). The distances between populations are ca. 420 km between Nord‐Pas‐de‐Calais and Indre, ca. 420 km between Nord‐Pas‐de‐Calais and Saarland, ca. 180 between Nord‐Pas‐de‐Calais and Zeeland, ca. 350 km between Saarland and Zeeland, and ca. 550 km between Indre and Saarland and between Indre and Zeeland. Despite the relatively small spatial scale, the edge populations are clearly differentiated from each other and from the core populations as indicated by neutral genetic markers (Swaegers et al. 2016). Moreover, common‐garden rearing experiments from the egg stage showed the evolution of a faster life history (Therry et al. 2014b) and a higher investment in flight muscles and immune response (Therry et al. 2014c) in the edge populations at the range front compared to the nearby core populations.

The study populations at Nord‐Pas‐de‐Calais, Indre, and Zeeland are in natural areas without agriculture and therefore were unlikely to be affected by pesticides (Coors et al. 2009; Cothran et al. 2013). The edge population in Saarland is within an agricultural area. This could have affected our results in two opposing ways: (i) animals in the Saarland population may have developed tolerance to the pesticide (e.g., Hua et al. 2015), or (ii) animals in the Saarland population may have suffered stress in the parental generation due to contamination of the habitat making them more vulnerable to pesticide exposure in the laboratory. The local adaptation option is unlikely given that polluted, and unpolluted ponds are intermixed in the landscape and the high levels of gene flow at a regional scale in *Coenagrion* damselflies (Johansson et al. 2013) and given the Saarland population was founded recently when sampled (<5 years, Therry et al. 2014c). Also, any local adaptation to pesticides in this edge population would make our results of increased vulnerability to pesticides in edge populations conservative. Furthermore, we did not detect differential effects of the pesticide on any response variable between the Saarland edge population and the other edge population in Zeeland, which was situated in a natural area (Appendix S1). This also suggests that any effects on the experimental larvae in the Saarland population working through stress due to pesticide contamination of the habitat is unlikely as it would have generated a higher vulnerability in the Saarland compared to the Zeeland edge population.

Mated females (Nord‐Pas‐de‐Calais: 8, Indre: 12, Saarland: 12, and Zeeland: 11) were collected in June–July 2012 and allowed to oviposit *in situ*. Eggs were transported to the laboratory in Belgium. After hatching, larvae of each female were kept together in a plastic tank (15 × 10 × 12.5 cm) filled with ca. 500 mL dechlorinated tap water for 3 weeks to enhance survival. During this period, larvae were kept at 20°C and a photoperiod of 16:8 h light:dark. Larvae were fed *Artemia* nauplii *ad libitum* 5 days per week. After this 3‐week period larvae were introduced in the container experiment.

### Outdoor container experiment

To test whether evolutionary processes during the range expansion affect the vulnerability of *Coenagrion scitulum* damselflies to a pesticide and how density may play a role in modifying these effects, we set up a full factorial outdoor container experiment with 2 population types (edge and core, each represented by two populations) × 2 densities (low and high) × 3 esfenvalerate concentrations (0, 0.1 and 0.2 μg/L). Each treatment combination had 8 replicated containers (10 L polypropylene cylindrical tanks, height of 22 cm, diameter of 24 cm) giving a total of 96 containers. The container experiment consisted of three periods: (i) a pre‐exposure period that started when larvae were ca. 3 weeks old and that spanned fall and winter, (ii) a pesticide exposure period of 4 weeks in spring during which the larvae experienced four pulses of esfenvalerate, and (iii) a postexposure period that ended with adult metamorphosis. The initially installed larval densities were 15 and 45 larvae per container, corresponding to low (332 larvae per m^2^) and high (995 larvae per m^2^) densities of coenagrionid damselfly larvae in suitable habitats (Corbet 1999), respectively.

Due to higher mortality in the pre‐exposure stage of the experiment in edge (62.89%) than in core populations (58.1%) (Loglinear model, $\chi_{1}^{2}$ = 6.88, *P *<* *0.0088) and in high‐density (64.10%) than in low‐density containers (49.13%) (Loglinear model, $\chi_{1}^{2}$ = 48.15, *P *<* *0.001) and the resulting density variation among containers of the same density treatment, we re‐installed the density treatments after winter. This was carried out by redistributing larvae among containers (cf. Liess et al. 2013), thereby keeping larvae at their combination of population and density. Note this was carried out just before the pesticide exposure period started. The new densities were 8 (low density) and 20 (high density) larvae per container. The resulting number of containers per density treatment varied from 5 to 8 per population (exact numbers are shown in the figures). See Appendix S2 for more details of the experimental setup.

### Application of esfenvalerate

The esfenvalerate concentrations were chosen based on a 48 h acute toxicity test in which *C. scitulum* damselfly larvae were individually exposed to concentrations of 0, 0.25, 0.5, 1, and 2 μg/L at 18°C (close to the temperature in the containers at the start of the exposure period, see Fig. S1G–H). After 48 h the survival was 100% in the control, 82% at 0.25 μg/L, 33% at 0.5 μg/L and 0% at 1 and 2 μg/L. In another acute toxicity test in which *Daphnia pulex*, the food source of the damselfly larvae in the containers, was exposed in groups of 10 individuals to the same esfenvalerate concentrations at 18°C, none of the *Daphnia* died after 48 h, even at the highest tested esfenvalerate concentration of 2.0 μg/L. Because we were also interested in sublethal effects, we selected concentrations of 0.1 μg/L (the lowest observed effect concentration for invertebrates, European Commission 2000) and 0.2 μg/L (below the lowest lethal concentration in our acute toxicity test) for our experiment. Both concentrations fall within the range of concentrations found in water bodies nearby agricultural areas, which go up to 0.76 μg/L (Stampfli et al. 2013). A 1 mg/mL stock solution was prepared by dissolving esfenvalerate powder (purity >99%, Sigma‐Aldrich) in absolute ethanol. This stock solution was further diluted with filtered water from the containers to obtain concentrations of 12 and 24 μg/L esfenvalerate (the spraying solutions), respectively. Fifty millilitre of each spraying solution (12 and 24 μg/L) was gently poured over the surface of the containers (Stampfli et al. 2013) to obtain the nominal esfenvalerate concentrations of 0.1 and 0.2 μg/L. In the control treatment, we added 50 mL ethanol (24 μL/L), using the ethanol concentration of the high esfenvalerate treatment, in the same manner as in the pesticide treatments.

Esfenvalerate was applied four times in spring, with 1 week between pulses, starting on 2 May 2013 with the last pulse on 23 May 2013. This mimics the realistic scenario of exposure to several pesticide pulses in spring through run‐off (Van Drooge et al. 2001). The measured concentrations in the containers, based on a pooled sample from all containers of each of the exposure concentrations, were 0.072 and 0.084 μg/L 2 h after spraying (the expected peak concentration, Knillmann et al. 2013), for the nominal concentrations of 0.1 and 0.2 μg/L, respectively. After 1 week, just before applying a new pulse, the concentrations were below the detection limit of 0.005 μg/L. Esfenvalerate concentrations were analyzed by the research laboratory Lovap NV, Geel, Belgium using gas chromatography in combination with mass spectrometry.

### Abiotic and biotic parameters

Temperature, dissolved oxygen, pH, and conductivity were measured in a subsample of 24 containers, 2 containers per combination of population type × density × esfenvalerate concentration. These parameters were quantified biweekly throughout the exposure and postexposure periods. Chlorophyll *a* concentrations were measured in all containers on a biweekly basis during the exposure and postexposure periods. The abundance of *D. pulex* was quantified in each container at the start of the pesticide exposure period to obtain the initial density, and after 7 days to obtain the lowest density. Thereafter, *Daphnia* abundance was quantified every 2 weeks just before (lowest density) and after (highest density) the weekly addition of *Daphnia*. See Appendix S2 for detailed overviews of the temporal patterns of abiotic and biotic parameters in the experimental containers under the different treatment combinations.

### Response variables

To estimate larval growth rate during the 4‐week exposure period, all larvae from each container were collected and weighted on 26–29 April 2013 (just before the start of the exposure period) and on 29–30 May 2013 (end of the exposure period). Mean per capita mass per container was used to calculate growth rate as (ln_final mass_ – ln_initial mass_)/duration exposure period. Based on the number of larvae counted at the start and at the end of the exposure period, we calculated mortality (%) during the exposure period as (initial number – number of survived larvae)/initial number of larvae × 100. After the exposure period, we daily checked for emergence of adult damselflies. The larval development time was calculated as the time from egg hatching to adult emergence. To quantify mass at emergence, all freshly‐emerged adults were kept in the dark for ca. 16 h to harden their exoskeleton where after their wet mass was weighted to the nearest 0.01 mg using an electronic balance (AB135‐S, Mettler Toledo^®^, Zaventem, Belgium). Afterward, all adults were stored at −80°C until the analyses of flight muscle mass and fat content. For each adult that emerged, flight muscle mass and fat content, two important flight‐related traits (Therry et al. 2014c), were quantified based on protocols described in Swillen et al. (2009) (see Appendix S3 for more detail).

### Statistical analyses

To test for effects of the population type, larval density, and esfenvalerate concentration on the response variables mortality, growth rate, and development time during the exposure period, and adult mass at emergence, flight muscle mass, and total fat content, we ran separate an(c)ovas using the mixed procedure of SAS v9.3 (SAS Institute Inc., Cary, NC, USA). In all models, population nested in population type was included as a random factor. When testing effects on flight muscle mass and fat content, we included the exoskeleton mass as covariate to correct for size differences (see Therry et al. 2014c). All models use containers as the unit of replication. We will here report results for total development time, the patterns for the duration of the postexposure period (relevant for potential recovery) are similar and shown in Appendix S4.

In damselflies, sexes may differ in their response to pesticide exposure (e.g., Campero et al. 2008). We therefore sexed all adults at emergence and analyzed the traits scored at emergence separately by sex (development time, mass at emergence, flight muscle mass, and fat content). Note that given the large number of larvae (>1000 larvae) involved, it was logistically not possible to sex all larvae at the start and the end of the exposure period, so we could not separately analyze larval traits by sex.

## Results

### Larval life history traits

Surprisingly, in total 71 adults emerged before winter during the months of October and November 2012, hence before the pesticide exposure period started. These were all edge animals (anova on numbers emerged per container, Population type: *F*_1,92_ = 26.35, *P *<* *0.0001), and numbers did not differ between containers at low density (*n* = 28 adults) and containers at high density (*n* = 43 adults) (Density: *F*_1,92_ = 1.18, *P *=* *0.28).

**Figure 1:**
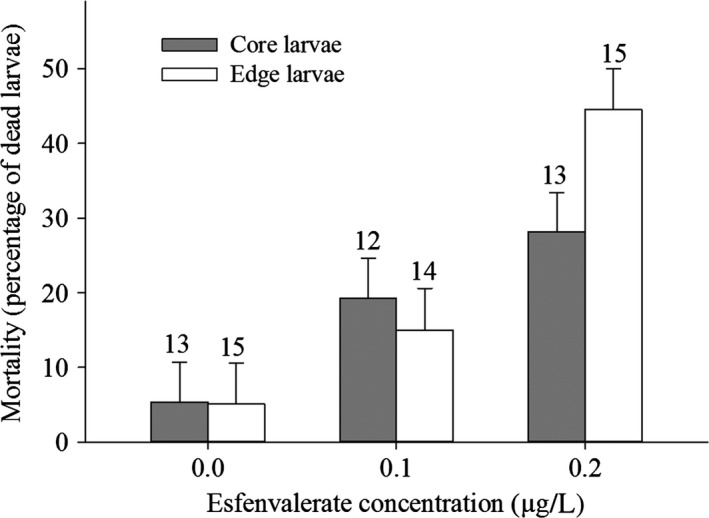
Mortality of *Coenagrion scitulum* damselfly larvae during the exposure period as a function of esfenvalerate concentration and population type. Numbers above the bars represent the number of container replicates. Least‐square means are given with 1 SE.

Overall mortality during the spring exposure period did not differ between edge and core populations (*F*_1,2_ = 0.4, *P *=* *0.59) and between low and high density (*F*_1,68_ = 1.13, *P *=* *0.29). Exposure to esfenvalerate increased mortality (*F*_2,68_ = 34.34, *P *<* *0.001). Notably, the effect of esfenvalerate differed between edge and core populations (Population type × Pesticide, *F*_2,68_ = 4.1, *P *=* *0.021). Contrasts analyses showed that at the high esfenvalerate concentration the pesticide‐induced mortality was stronger in edge populations than in core populations (*F*_1,70_ = 8.03, *P *=* *0.006, Fig. 1), while mortality did not differ between edge and core populations in the control (*F*_1,70_ < 0.01, *P *=* *0.99) and at the low concentration (*F*_1,70_ = 0.37, *P *=* *0.54). This pattern of increased mortality of edge compared to core populations at the high concentration was similar at both densities (Population type × Pesticide × Density: *F*_2,68_ = 0.75, *P *=* *0.48, Fig. S4A,B in Appendix S5). The pesticide effect did not depend upon density (Density × Pesticide, *F*_2,68_ = 1.29, *P *=* *0.28).

Growth rates differed neither between edge and core populations (*F*_1,2_ = 0.02, *P *=* *0.91 Fig. S4C,D) nor between low and high densities (*F*_1,68_ = 0.2, *P *=* *0.65). Growth rate during the exposure period strongly decreased with increasing esfenvalerate concentrations: the growth reductions were ca. 27% at the low and ca. 36% at the high esfenvalerate concentration (*F*_2,68_ = 7.03, *P *=* *0.0017, Fig. S4C,D in Appendix S5). The pesticide effect did not depend upon population type or density (all interactions: *P *>* *0.25).

**Figure 2:**
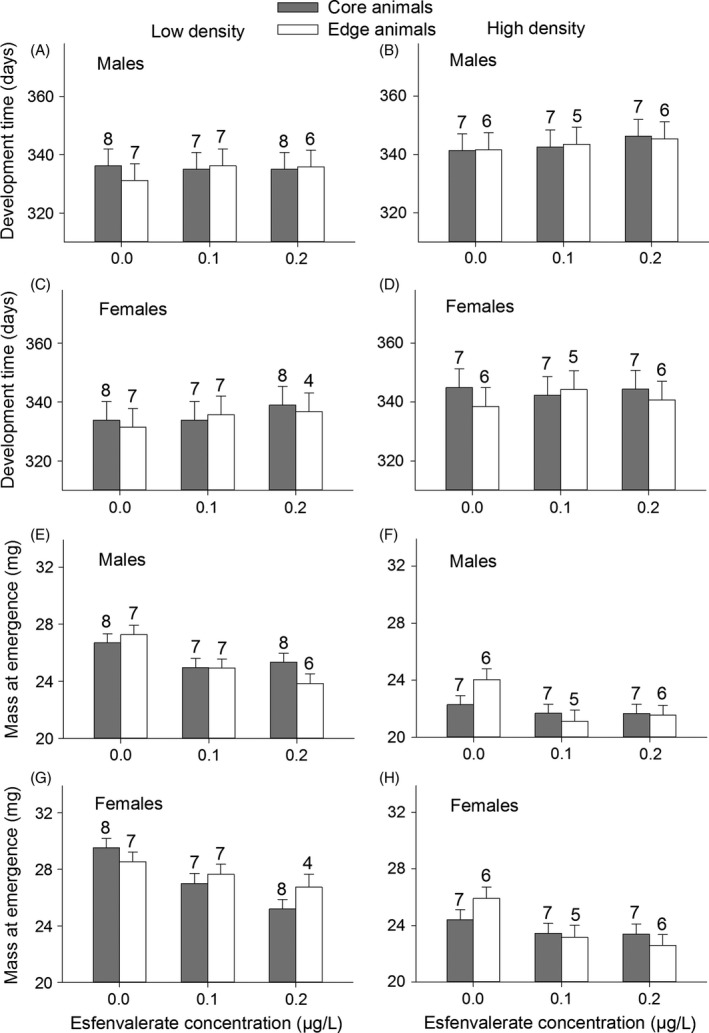
Development time of males (A, B) and females (C, D) and mass at emergence of males (E, F) and females (G, H) of the damselfly *Coenagrion scitulum* as a function of esfenvalerate concentration, density, and population type. Numbers above the bars represent the number of container replicates. Least‐square means are given with 1 SE.

Exposure to esfenvalerate tended to result in a slightly later emergence of ca. 3 days (Males: *F*_2,67_ = 3.1, *P *=* *0.051; Females: *F*_2,66_ = 2.73, *P *=* *0.072, Fig. 2A,D). Development times were longer at high density (Males: *F*_1,67_ = 69.1, *P *<* *0.001; Females: *F*_1,66_ = 47.7, *P *<* *0.001, Fig. 2B,D). Development times tended to be slightly shorter in edge females than in core females in the control and at the high esfenvalerate concentration (Females: Population type × Pesticide, *F*_1,66_ = 3.12, *P *=* *0.051, Fig. 2C,D).

### Adult flight‐related traits

Mass at emergence decreased with increasing esfenvalerate concentrations (Males: *F*_2,67_ = 7.64, *P *=* *0.001; Females: *F*_2,65_ = 8.4, *P *<* *0.001, Fig. 2E–H) and was lower at high density (Males: *F*_1,67_ = 55.65, *P *<* *0.001; Females: *F*_1,65_ = 47.17, *P *<* *0.001). Mass at emergence did not differ between edge and core animals (Males: *F*_1,2_ = 0.04, *P *=* *0.86; Females: *F*_1,2_ = 0.08, *P *=* *0.80, Fig. 2E–H).

**Figure 3:**
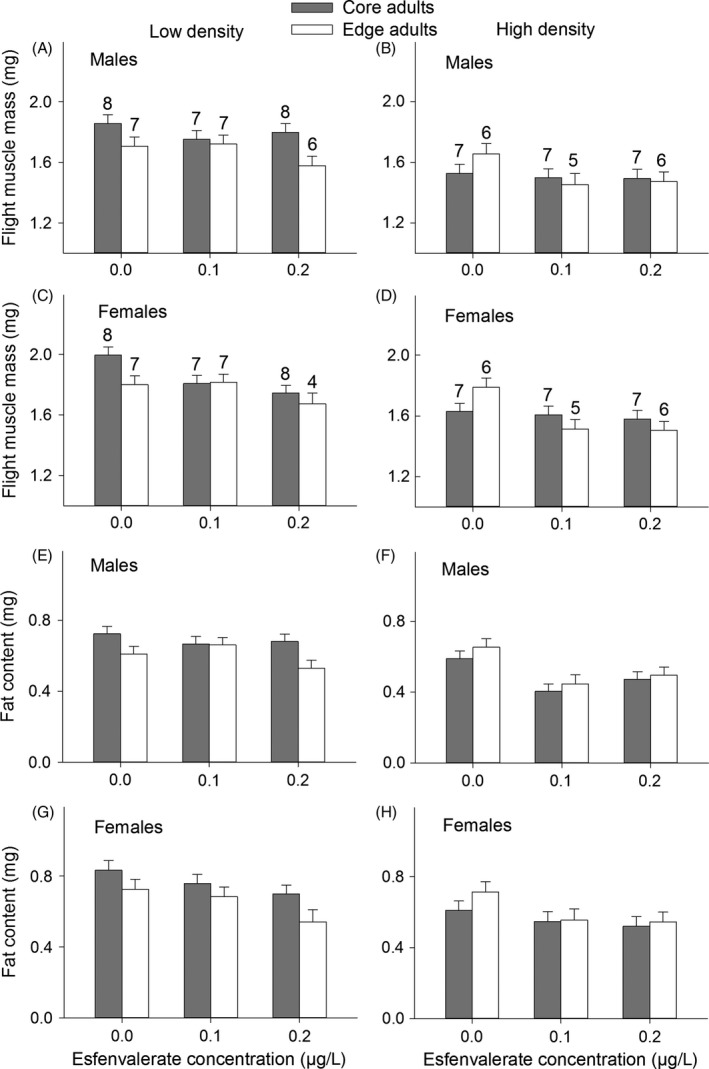
Flight muscle mass of males (A, B) and females (C, D), fat content of males (E, E) and females (G, H) of the damselfly *Coenagrion scitulum* as a function of esfenvalerate concentration, density, and population type. Numbers above the bars represent the number of container replicates. Least‐square means corrected for size are given with 1 SE.

Exposure to esfenvalerate negatively affected the relative flight muscle mass (Males: *F*_2,66_ = 2.79, *P *=* *0.068; Females: *F*_2,64_ = 9.42, *P *<* *0.001, Fig. 3A–D). Edge animals tended to have a higher flight muscle mass than core animals at high density in the absence of the pesticide, while the opposite was observed at low density (Males: Population type × Density, *F*_1,66_ = 4.75, *P *=* *0.033; Females: Population type × Density × Pesticide, *F*_2,64_ = 6.18, *P *=* *0.0035). High density resulted in a lower flight muscle mass (Males: *F*_1,66_ = 29.35, *P *<* *0.001; Females: *F*_1,64_ = 21.29, *P *<* *0.001).

Exposure to esfenvalerate strongly decreased the fat content (Males: *F*_2,66_ = 5.58, *P *=* *0.0058; Females: *F*_2,64_ = 7.61, *P *=* *0.0011). In males, this pesticide effect was density‐dependent (Density × Pesticide, *F*_2,66_ = 4.89, *P *=* *0.011, Fig. 3E–H): at low‐density fat content was only reduced at the high esfenvalerate concentration while at high‐density fat content was already reduced at the low esfenvalerate concentration. In both sexes, fat content was lower at high density than at low density (Males: *F*_1,66_ = 18.89, *P *<* *0.001; Females: *F*_1,64_ = 10.94, *P *=* *0.0015); this pattern was stronger in core animals than in edge animals (Population type × Density, Males: *F*_1,66_ = 8.06, *P *=* *0.006; Females: *F*_1,64_ = 7.07, *P *=* *0.0099, Fig. 3E–H).

## Discussion

### Main effects of the pesticide

We found strong negative effects of larval exposure to the ecologically realistic esfenvalerate concentrations on all studied traits not only in the larval but also in the adult stage, and this despite the long period (ca. 25–30 days, see Appendix S4) that larvae were able to recover from pesticide exposure. Esfenvalerate‐imposed mortality fits the pattern of lethal effects imposed by pyrethroids in aquatic insects (Liess 2002; Beketov and Liess 2005; Rasmussen et al. 2013), which result from damage to the nervous system (Cold and Forbes 2004). The negative effects of exposure to esfenvalerate on growth and flight‐related traits likely were mediated by energy shortage as pesticide‐exposed animals need more energy for detoxification and repair, resulting in less energy allocation toward other functions (Campero et al. 2007). Note that these esfenvalerate effects are likely direct effects on the damselfly larvae and no indirect effects working through the *Daphnia* food because *D. pulex* survival was not affected by a 10× higher esfenvalerate concentration and because the pesticide did not affect the abundance of *D. pulex* in the containers (Appendix S2).

A key finding was that esfenvalerate negatively affected body mass, fat content, and relative flight muscle mass, three traits known to shape flight performance in *Coenagrion* damselflies (Gyulavári et al. 2014; Therry et al. 2014c). Delayed effects of esfenvalerate across metamorphosis have also been documented in the caddisfly *Brachycentrus americanus* where adults that had been exposed to esfenvalerate in the pupal stage invested less in egg mass (Palmquist et al. 2008). The pesticide‐induced reductions in the flight‐related traits, especially flight muscle mass is a highly relevant trait for key functions such as flight ability (e.g., Therry et al. 2014a), and therefore important for shaping foraging, predator evasion, mating success, and dispersal ability in damselflies (Stoks and Cordoba‐Aguilar 2012).

### Edge‐core differentiation mediating the effect of the pesticide

We found some evidence for the expected faster life history and increased investment in flight morphology in edge populations. Animals at an expanding range are expected to show a faster life history because of spatial sorting and r‐selection associated with the initial lower population densities at the expansion front (Phillips 2009; Burton et al. 2010; Phillips et al. 2010). For the study species this also includes selection for a fast development to avoid having less generations per year at the higher latitudes at the expansion front (Nilsson‐Örtman et al. 2012) which would slow down the range expansion (Therry et al. 2014b). Therry et al. (2014b) indeed reported higher growth and development rates in edge larvae of the study species. In line with this, we observed that the subset of animals that were able to emerge before winter were all edge animals. Yet, within the subset of larvae that overwintered (hence those that were used in the spring exposure experiment), no faster life history in edge animals was observed. The latter may be a result of the fastest animals already emerging before winter. Moreover, the higher mortality during winter in edge populations (see methods) may have mainly removed the faster growing larvae. Indeed, rapid growth has been associated with reduced energy storage (Stoks et al. 2006) and reduced cold resistance (Stoks and De Block 2011) in damselflies, which may have reduced the ability to survive winter. More general, a faster life history has been associated with a higher mortality in damselfly larvae (De Block et al. 2008; Sniegula et al. 2014). Edge animals are also expected to have a higher relative flight muscle mass as only the best dispersers may reach the expansion front (Shine et al. 2011). Indeed, edge populations of poleward‐moving insects, including the study species (Therry et al. 2014c), show a higher investment in flight muscle mass (reviewed in Hill et al. 2011). Yet, in current study this was only observed at high density (in the control without the pesticide) suggesting that the higher investment in flight muscles in edge populations may be density‐dependent.

Our data suggested that edge animals had a higher vulnerability to the pesticide in term of a higher mortality at the high esfenvalerate concentration. Note that this higher vulnerability in edge populations did not occur at the low pesticide concentration as at the low concentration our contrast analysis suggested no significant difference of mortality between edge and core populations. Notably, we observed the higher vulnerability to the pesticide in edge populations despite no indication of a faster life history in the overwintered larvae that were exposed to the pesticide and without a consistent higher investment in flight morphology. Edge populations, however, are expected to show rapid evolutionary changes in a wide range of traits, including life history, morphology, behavior, and physiology (Phillips 2009; Burton et al. 2010; Phillips et al. 2010; Shine et al. 2011; Brown et al. 2015) that all require a higher allocation of energy and therefore are expected to be traded off against investment in detoxification and repair (Sibly and Calow 1989; Congdon et al. 2001). For example, the edge animals may have invested more in immune function to avoid parasite‐driven reductions in dispersal ability, as has been observed in the study species (Therry et al. 2014b; K. V. Dinh, L. Janssens, L. Therry, L. Bervoets and R. Stoks, unpublished data). Additionally, in another outdoor container experiment (L. Therry, and R. Stoks in prep.) edge larvae of the study species showed a faster growth during the winter period and as a result had a lower fat content after winter, which may have made them more vulnerable to the pesticide. Whatever the mechanism, our results suggest that evolutionary changes associated with range expansion, made edge populations more vulnerable to esfenvalerate during spring application. Admittedly, the increase in pesticide‐induced mortality in edge compared to core populations was relatively small (ca. 17%), yet will translate into extra reductions in population growth rates if edge populations are exposed to pesticides.

### Larval density mediating the effects of the pesticide

While the high‐density treatment did not influence larval survival and growth during the pesticide exposure period, larvae reared at high density showed longer larval development times, and reductions in mass at emergence, flight muscle mass, and fat content. These negative density effects are in line with a higher exploitation competition for food at the high‐density treatment. Additionally, at high densities there may have been more physical encounters among larvae, thereby imposing stress; this is especially likely in damselfly larvae as they are cannibalistic and impose predator stress on each other (De Block and Stoks 2004). Another important finding was that high density only reduced flight muscle mass in core adults but not in edge adults which is in line with the hypothesis of a stronger selection for flight performance in edge populations (Hill et al. 2011; Therry et al. 2014c). Negative effects of larval competition on adult flight muscle mass have not previously been documented and provide a rare empirical example of how the conditions encountered during the larval stage may shape the adult dispersal ability (Benard and McCauley 2008). In males, the pesticide‐induced reduction in fat content was stronger at high density than at low density; this is in line with the stronger negative effect of pesticides at higher density in other aquatic animals (e.g., Jones et al. 2011; Knillmann et al. 2012).

### Implications for ecological risk assessment and range expansions

Current ecological risk assessment (ERA) of pesticides is not effectively protecting biodiversity as strong losses in biodiversity are being detected at concentrations that current legislation considers as environmentally protective (Beketov et al. 2013; Malaj et al. 2014). Our study adds to this by identifying two reasons why current ERA may underestimate the impact of pesticides, and thereby points to concrete actions to improve legislation to make toxicity testing more effective toward management and protection of freshwater biodiversity under global warming. Firstly, we build further on previous insights that standard toxicity testing limited to one life stage may not capture the full impact of a pesticide (see, e.g., Campero et al. 2008; Distel and Boone 2010; Janssens et al. 2014). We thereby made an important extension by providing evidence that larval exposure to ecologically relevant concentrations of pesticides may negatively affect locomotory performance in the adult stage. This ignored delayed effect of pesticides may have major fitness consequences as locomotion is crucial for key functions such as foraging, escaping predation, securing matings, and dispersal (Stoks and Cordoba‐Aguilar 2012). Secondly, we provide the first test and some supporting evidence that edge populations at an expanding range front are more vulnerable to high pesticide concentrations than core populations in term of a higher pesticide‐induced mortality, thereby adding an evolutionary component to the emerging insight that we need spatially explicit ERA (Van den Brink 2008; Clements et al. 2012; Dinh Van et al. 2014).

Both the effect of larval pesticide exposure on mortality and its delayed effects on adult flight‐related traits also are highly relevant to understand the impact of global warming on organisms as they highlight two overlooked pathways of how pesticides may slow down range expansions. Firstly, exposure to esfenvalerate at ecologically realistic concentrations caused mortality and thereby decreases in population growth rates, hence it is expected to reduce the rate of further range expansion. In addition, our data indicated rapid evolution of a slightly increased pesticide‐induced mortality at the range front, which has the potential to magnify this effect, and thereby to slow down the range expansion even more. As species may show considerable population declines in core regions under global warming, researchers highlighted the need for direct conservation efforts toward leading‐edge populations for spearheading future range shifts (Razgour et al. 2013). Our results thereby underscore the importance of considering pesticide exposure in such conservation programs. Secondly, esfenvalerate negatively affected three flight‐related traits (body mass, relative flight muscle mass, and fat content) known to shape flight performance in *Coenagrion* damselflies (Gyulavári et al. 2014; Therry et al. 2014c), thereby reducing the dispersal ability. Any reductions in dispersal rates may have major implications as there is increasing concern that poleward range expansions do not allow timely tracking of the moving climate niche (La Sorte and Jetz 2012). These two overlooked mechanisms how pesticides may slow down range expansion, together with the expected increase in pesticide application at higher latitudes under global warming (Kattwinkel et al. 2011), raise concern about the potential for edge populations to act as potent sources for further range expansion in a polluted world.

Despite recent progress in identifying factors underlying species differences in range expansion rates (Angert et al. 2011; Mair et al. 2014), it is largely unknown why there is so much variation in the rates at which different species’ geographic ranges expand in response to climate warming (Moritz and Agudo 2013). Yet, this information is crucial to identify species that may potentially be too slow to track their moving climate niche, thereby being more at risk under global warming and to understand the likely success of different conservation strategies for facilitating range shifts (Moritz and Agudo 2013; Mair et al. 2014). Some of the current models predicting future ranges already include estimates of dispersal ability to predict which species may be better at tracking their climate envelope (e.g., Thomas et al. 2001; Hughes et al. 2007). Species may differ considerably in their sensitivity to pesticides (e.g., Beketov 2004; Rasmussen et al. 2013; Weston et al. 2013). Our results therefore generate the hypothesis that besides dispersal ability also the degree to which survival and dispersal ability are affected by widely used contaminants and how the vulnerability to pesticides evolves at expanding range fronts may be key factors in shaping species differences in range expansion in a polluted world.

## Data accessibility

Data for this study are available at the Dryad Digital Repository: DOI: doi:10.5061/dryad.cb978.

## Supporting information


**Appendix S1.** Testing for potential of local pesticide adaptation in the Saarland edge population.
**Table S1.** The results for the Population × Pesticide interaction in the ANC(O)VAs testing for the effects of population and pesticide on the measured response variables within the set of two studied edge populations of the damselfly *Coenagrion scitulum*.

**Appendix S2.** Outdoor container experiment.
**Figure S1.** Means of electrical conductivity (A, B), pH (C, D), dissolved oxygen (E, F) and temperature (G, H) in the experimental containers as a function of larval density and esfenvalerate concentration.
**Figure S2.** Means of chlorophyll *a* (A, B), and *Daphnia* abundance (C, D) in the experimental containers as a function of larval density and esfenvalerate concentration.

**Appendix S3.** Quantification of flight muscle mass and fat content.

**Appendix S4**. Duration of post‐exposure period.
**Figure S3**. Duration of the post‐exposure period of males (A, B) and females (C, D) of the damselfly *Coenagrion scitulum* as a function of esfenvalerate concentration, density and population type.

**Appendix S5.** Mortality and growth rate of damselfly larvae during the exposure period.
**Figure S4.** Mortality (A, B) and growth rate (C, D) of *Coenagrion scitulum* damselfly larvae during the exposure period as a function of esfenvalerate concentration, density and population type.
