# Supplementary material for: Rapid evolution of increased vulnerability to an insecticide at the expansion front in a poleward‐moving damselfly
Source: Evol Appl. 2016 Jan 27;9(3):450–61. doi: 10.1111/eva.12347 (PMC4778112; doi:10.1111/eva.12347)
Supplement: Supplementary file 1 — Appendix S1. Testing for potential of local pesticide adaptation in the Saarland edge population. Table S1. The results for the Population × Pesticide interaction in the ANC(O)VAs testing for the effects of population and pesticide on the measured response variables within the set of two studied edge populations of the damselfly Coenagrion scitulum. [file EVA-9-450-s001.docx]

**Appendix S1. Testing for potential of local pesticide adaptation in the Saarland edge population**

To evaluate the presence of local adaptation to pesticides and the potential additional stress due to contamination of the habitat working through the maternal effects on larvae in the Saarland edge population (the only population situated in agricultural land) we specifically tested whether it reacted differently to the pesticide than the another edge population for any of the variables measured (mortality, growth rate, development time, mass at emergence, relative flight muscle mass and fat content). We therefore ran separated ANC(O)VAs per response variable on the set of two edge populations and included population, density and pesticide as fixed factors. In case of local pesticide adaptation or additional stress due to contamination of the habitat in the Saarland population this would translate in a significant Population × Pesticide interaction. When testing effects on relative flight muscle mass and fat content, we included the exoskeleton mass as covariate to correct for size differences (see [Therry et al. 2014](#_ENREF_2)). All models used containers as the unit of replication.

In damselflies, sexes may differ in their response to pesticide exposure (e.g., [Campero et al. 2008](#_ENREF_1)). We therefore sexed all adults at emergence and analysed the traits scored at emergence separately by sex (development time, mass at emergence, flight muscle mass and fat content). Note that it is not possible to sex animals before the final instar, so we could not separately analyse larval traits by sex.

**Results and discussion**

None of the measured variables (Table S1) showed a significant Population × Pesticide interaction, indicating that both edge populations responded similarly to the pesticide. This argues against the presence of local pesticide adaptation or additional stress due to contamination of the habitat in the Saarland edge population.

Table S1. The results for the Population × Pesticide interaction in the ANC(O)VAs testing for the effects of population and pesticide on the measured response variables within the set of two studied edge populations of the damselfly *Coenagrion scitulum*.

| Response variable | df1, df2 | F | *P* |
| --- | --- | --- | --- |
| Mortality | 2, 26 | 1.21 | 0.31 |
| Growth rate | 2, 26 | 0.03 | 0.97 |
| Development time of males | 1, 25 | 1.69 | 0.20 |
| Development time of females | 1, 24 | 1.25 | 0.30 |
| Mass at emergence of males | 2, 25 | 0.08 | 0.93 |
| Mass at emergence of females | 2, 23 | 0.43 | 0.66 |
| Relative flight muscle of males | 2, 24 | 0.074 | 0.93 |
| Relative flight muscle of females | 2, 22 | 0.97 | 0.39 |
| Fat content of males | 2, 24 | 1.02 | 0.38 |
| Fat content of females | 2, 22 | 0.23 | 0.80 |

**Literature cited**

Campero, M., M. De Block, F. Ollevier, and R. Stoks. 2008. Correcting the short-term effect of food deprivation in a damselfly: mechanisms and costs. *Journal of Animal Ecology* **77** (1):66-73.

Therry, L., V. Nilsson-Ortman, D. Bonte, and R. Stoks. 2014. Rapid evolution of larval life history, adult immune function and flight muscles in a poleward-moving damselfly. *Journal of Evolutionary Biology* **27** (1):141-152.
