# Supplementary material for: Rapid evolution of increased vulnerability to an insecticide at the expansion front in a poleward‐moving damselfly
Source: Evol Appl. 2016 Jan 27;9(3):450–61. doi: 10.1111/eva.12347 (PMC4778112; doi:10.1111/eva.12347)
Supplement: Supplementary file 2 — Appendix S2. Outdoor container experiment. Figure S1. Means of electrical conductivity (A, B), pH (C, D), dissolved oxygen (E, F) and temperature (G, H) in the experimental containers as a function of larval density and esfenvalerate concentration. Figure S2. Means of chlorophyll a (A, B), and Daphnia abundance (C, D) in the experimental containers as a function of larval density and esfenvalerate concentration. [file EVA-9-450-s002.docx]

**Appendix S2. Outdoor container experiment**

The experiment was run in outdoor 10 L polypropylene containers (height of 22 cm, diameter of 24 cm). Containers were installed at an outdoor experimental area (+ 50°51’N, + 4°40’E) in Heverlee (Belgium) and filled with 3 L dechlorinated tap water mixed with 3 L of water from an adjacent pond not surrounded by agriculture. Each container was covered with a net to prevent predators entering and adult damselflies escaping. We inoculated each container with ca. 500 *Daphnia pulex* and added ca. 50 mg grass to stimulate growth of protozoa. The containers were set up 7 days before the introduction of the damselfly larvae, thereby allowing the growth of *Daphnia* and protozoan populations as food for the small damselfly larvae. To avoid food depletion and to maintain similar food levels across containers during the pre-exposure period, we weekly added size-sorted *Daphnia pulex* (see below) to obtain a density of ca. 500 individuals per container. *Daphnia* were obtained from ten large 1000 L outdoor stock tanks. During the exposure period we weekly added ca. 600 - 700 *Daphnia* to all containers, irrespective of the density × pesticide treatment. This implied that gradually more food was present in low-density containers than in high-density containers (Fig. S2C-D), thereby further enforcing food exploitation competition in the high-density containers.

At the start of the experiment we installed two larval densities: 15 or 45 larvae per container, corresponding to low (332 larvae per m²) and high (995 larvae per m²) densities, respectively. Larval densities in the containers correspond to typical field densities of coenagrionid damselfly larvae in suitable habitats ([Corbet 1999](#_ENREF_1)). Larvae from the different females per population were randomly distributed among all containers of both density treatments of that population. Due to higher mortality in the pre-exposure stage of the experiment in edge (62.89%) than in core populations (58.1%) (Loglinear model, *χ*²_1_ = 6.88, *P* < 0.0088) and in high-density (64.10%) than in low-density containers (49.13%) (Loglinear model, *χ*²_1_ = 48.15, *P* < 0.001) and the resulting density variation among containers of the same density treatment, we re-installed the density treatments after winter. This was done by redistributing larvae among containers (cf. [Liess et al*.* 2013](#_ENREF_3)) thereby keeping larvae at their combination of population and density. Note this was done just before the pesticide exposure period started. The new densities were 8 (low density) and 20 (high density) larvae per container. The resulting number of containers per density treatment varied from 5 to 8 per population.

Conductivity, pH, dissolved oxygen, and temperature were biweekly measured in a subsample of 24 containers, 2 containers per combination of population type × density × esfenvalerate concentration. Conductivity (µS) and pH were measured with a multimeter (Multiline P4, WTW, Weilheim, Germany), dissolved oxygen (mg/l) with an oxygen electrode (HQ40d, Hach, Loveland, Co, USA) and temperature (°C) with a thermometer (Testo 926, Testo AG, Germany). All measurements were carried out in the afternoon (between 1 pm and 5 pm) in the center of the container at a depth of ca. 10 cm where most of damselfly larvae occurred.

Chlorophyll a concentrations were measured using a fluorometer (Turner Designs, Sunnyvale, CA, USA) in all containers on a biweekly basis during the exposure and post-exposure periods. Each time, 10 ml water from each container was sampled, kept at 4°C in the dark and the chlorophyll *a* concentrations were measured in triplicate the next morning (between 9 am and 11 am).

The abundance of *Daphnia pulex*, the food source of damselfly larvae in the containers, was quantified in each container at the start of the pesticide exposure period to obtain the initial density, and after 7 days to obtain the lowest density. Thereafter, abundance of *Daphnia pulex* was quantified every two weeks just before (lowest density) and after (highest density) the weekly addition of ca. 600-700 *Daphnia* individuals per container. To quantify *Daphnia* abundance, we gently mixed water in the containers to distribute *Daphnia* evenly. Subsequently, we swept a net (size 10 × 7 cm and mesh size 500 µm) once halfway through the water column along the diameter of the container. Pre-trials showed that in this way, ca. 25% of the *Daphnia* in a container were collected. The number of *Daphnia* was immediately counted, and all *Daphnia* returned to their containers.

To test whether the treatments affect the measured (a)biotic parameters in the containers, we ran separate repeated-measures ANOVAs with population type, density and pesticide concentration as fixed factors. We considered biweekly measurements of a given parameter in the same container as repeats. Population nested in population type was initially included as a random factor, yet it had no effect on any of the (a)biotic parameters and we removed it from the final models. *Daphnia* abundance and chlorophyll a concentrations were log(x+1)-transformed to meet ANOVA assumptions. As there was neither a significant main effect of population type nor its interaction with density and/or pesticide on the (a)biotic parameters we will not present population type in figures S1 and S2 to keep them informative. To further aid visualization we did not plot the standard errors of the container means in figures S1 and S2.

**Results and discussion**

The measured physiochemical parameters (conductivity, pH, dissolved oxygen, and temperature) changed through time (main effect Time, all *P* values < 0.001) but were largely independent of the pesticide treatment, density and population type (Figure S1). On average, conductivity was rather low and decreased from ca. 260 µS at the start of the experiment to ca. 180 µS at the end. pH values and dissolved oxygen levels were high and fluctuated between 9.4 and 10.5 (pH) and 11.2 and 16.1 mg/l (oxygen). Temperatures increased from ca. 19°C at the start of the experiment to ca. 25°C at the end.

On average, chlorophyll *a* concentrations were lower in the low density treatment (Density, F_1, 69_ = 9.13, *P* = 0.0035). Overall, this pattern was stronger through time (Time: F_3, 207_ = 22.31, *P* < 0.001; Time × Density: F_3, 207_ = 2.73, *P* = 0.045, Fig. S2A-B). The changes in chlorophyll *a* concentrations were probably associated with the higher *Daphnia* abundance at the low density treatment (see below) as *Daphnia* are important grazers on algae ([Feuchtmayr et al*.* 2010](#_ENREF_2)).

*Daphnia* abundance showed strong fluctuations with peaks reflecting the moments of weekly additions (see methods) followed by lower values due to predation by the damselfly larvae. Pesticide exposure had no effect on the Daphnia abundance (F_2, 70_ = 1.26, *P* = 0.29, Fig. S2C-D), indicating that food levels of damselfly larvae did not differ between the pesticide treatment and the control. In line with a role of predation by damselfly larvae in shaping *Daphnia* abundance, *Daphnia* was more abundant at the low density than at the high density treatment (Density, F_1, 70_ = 117.53, *P* < 0.001). This density effect became stronger through time likely due to higher predation rates by the growing damselfly larvae and the weekly addition of equal numbers of *Daphnia* at low and high density containers (Time, F_6, 420_ = 336.32, *P* < 0.001; and Time × Density, F_6, 420_ = 44.99, *P* < 0.001, Fig. S2C-D).

**
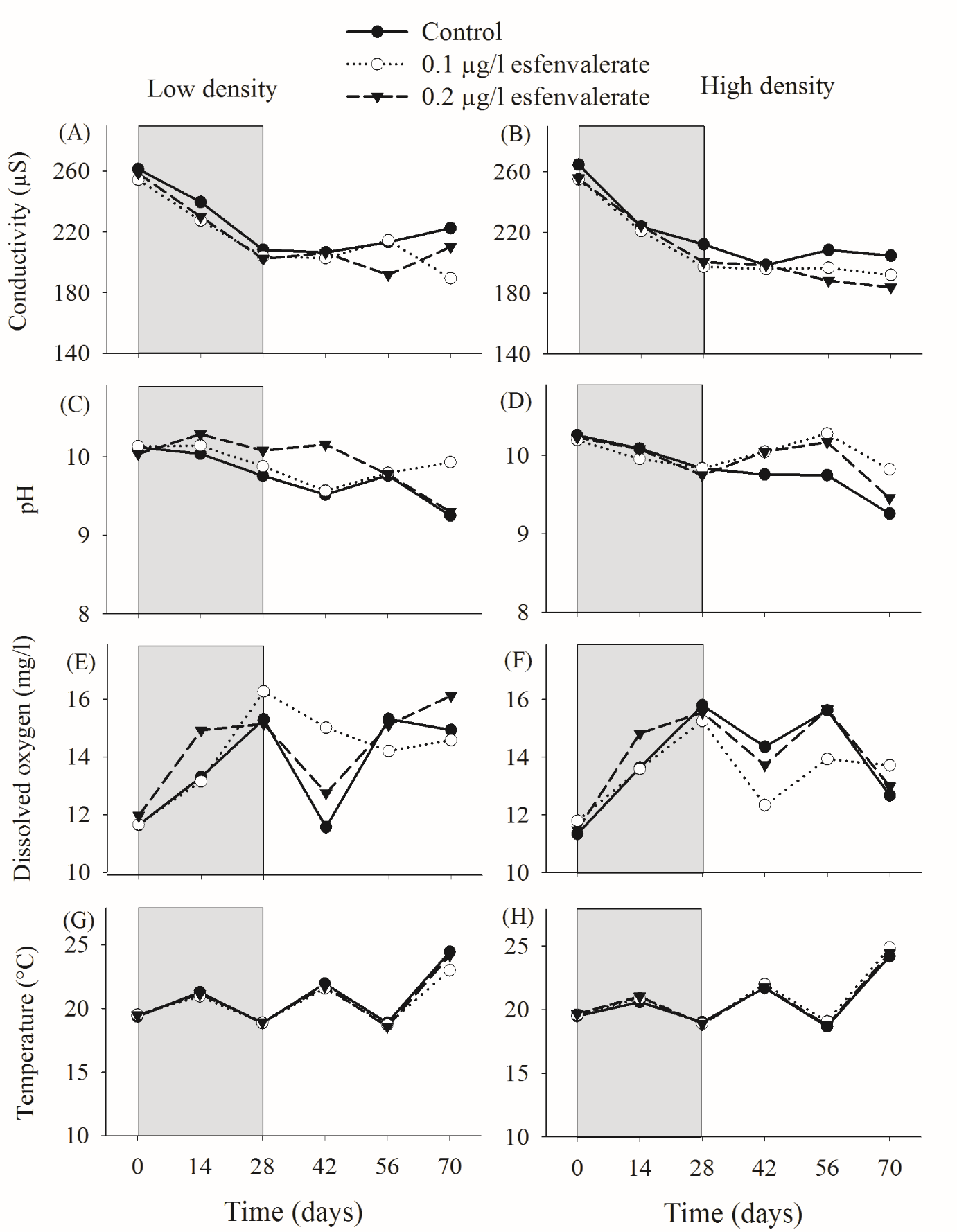
**

**Figure S1.** Means of electrical conductivity (A, B), pH (C, D), dissolved oxygen (E, F) and temperature (G, H) in the experimental containers as a function of larval density and esfenvalerate concentration. The pesticide exposure period is indicated in grey and started at day zero.


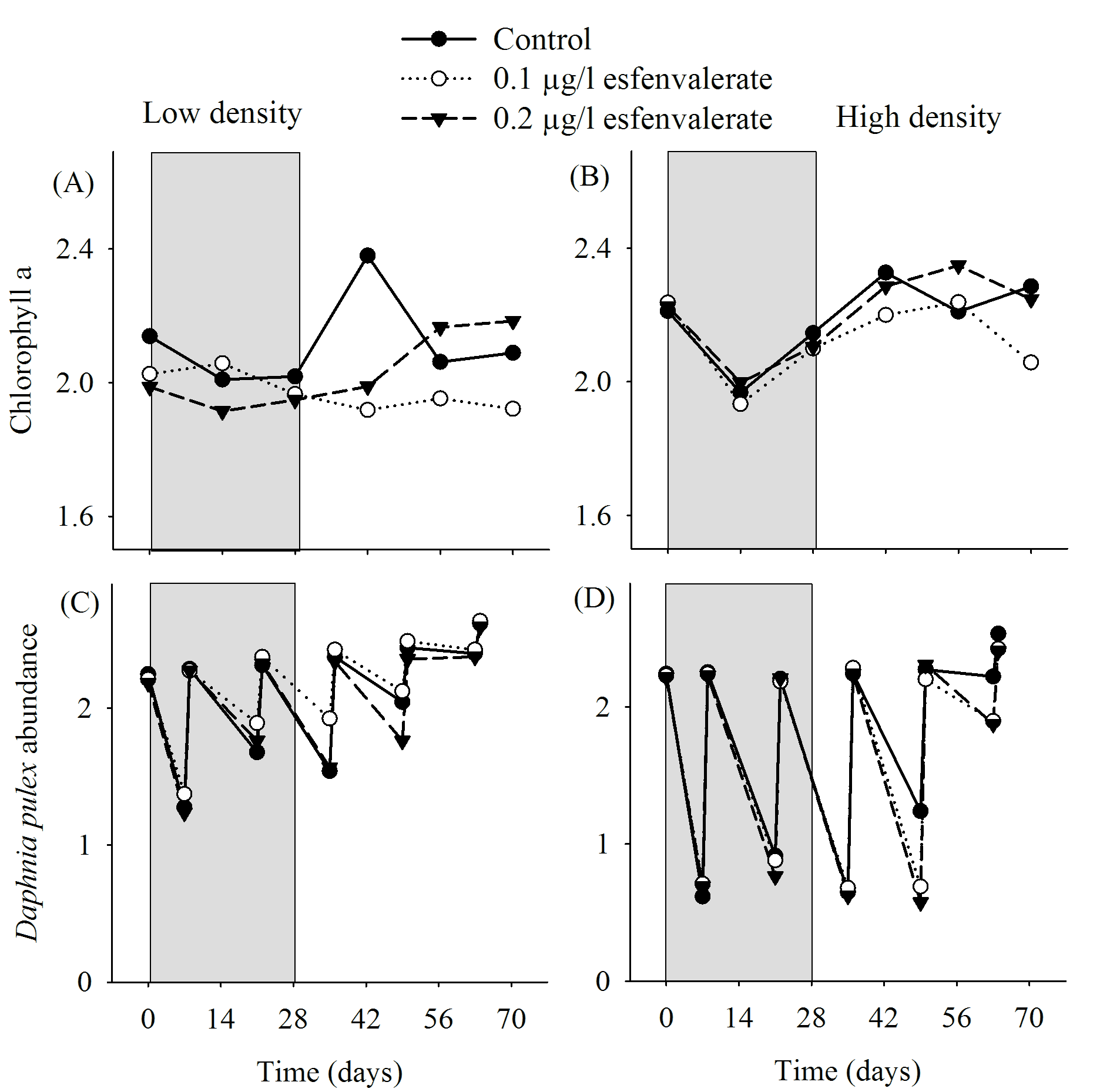


**Figure S2.** Means of chlorophyll *a* (A, B), and *Daphnia* abundance (C, D) in the experimental containers as a function of larval density and esfenvalerate concentration. The pesticide exposure period is indicated in grey and started at day zero. Given are means of log(x + 1)-transformed data.

**Literature cited**

Corbet, P. 1999. *Dragonflies: Behavior and ecology of Odonata*. London: Cornell University Press.

Feuchtmayr, H., B. Moss, I. Harvey, R. Moran, K. Hatton, L. Connor, and D. Atkinson. 2010. Differential effects of warming and nutrient loading on the timing and size of the spring zooplankton peak: an experimental approach with hypertrophic freshwater mesocosms. *Journal of Plankton Research* **32** (12):1715-1725.

Liess, M., K. Foit, A. Becker, E. Hassold, I. Dolciotti, M. Kattwinkel, and S. Duquesne. 2013. Culmination of low-dose pesticide effects. *Environmental Science & Technology* **47** (15):8862-8868.
