# Supplementary material for: Rapid evolution of increased vulnerability to an insecticide at the expansion front in a poleward‐moving damselfly
Source: Evol Appl. 2016 Jan 27;9(3):450–61. doi: 10.1111/eva.12347 (PMC4778112; doi:10.1111/eva.12347)
Supplement: Supplementary file 3 — Appendix S3. Quantification of flight muscle mass and fat content. [file EVA-9-450-s003.docx]

**Appendix S3. Quantification of flight muscle mass and fat content**

To quantify flight muscle and fat content, we first removed wings and legs and separated the head, thorax and abdomen using scissors. Thoraces and abdomens were placed individually in Eppendorf tubes, were dried for 48h at 60°C and were weighted separately to the nearest 0.01 mg using an electronic balance (AB135-S, Mettler Toledo^®^, Zaventem, Belgium). Subsequently, fat was extracted by adding 1.5 ml dichloromethane (99%) to the Eppendorf tubes which were placed for 24h on an automatic shaker. Thereafter, the dichloromethane with the dissolved fat was removed and the body parts were dried for another 48h at 60°C and weighted. Fat content was calculated by subtracting the dry mass after extraction from the dry mass before extraction. To obtain the flight muscle mass, we added 1.5 ml NaOH (0.35 M) to the Eppendorf tubes to break down all muscle tissue in the thorax samples, and placed the tubes on a shaker for 24h. The exoskeleton of thorax and abdomen were dried for another 48h at 60°C and weighted. Flight muscle mass was calculated by subtracting the thoracic exoskeleton mass from the total fatless thorax mass.
