# Supplementary material for: Rapid evolution of increased vulnerability to an insecticide at the expansion front in a poleward‐moving damselfly
Source: Evol Appl. 2016 Jan 27;9(3):450–61. doi: 10.1111/eva.12347 (PMC4778112; doi:10.1111/eva.12347)
Supplement: Supplementary file 4 — Appendix S4. Duration of post‐exposure period. Figure S3. Duration of the post‐exposure period of males (A, B) and females (C, D) of the damselfly Coenagrion scitulum as a function of esfenvalerate concentration, density and population type. [file EVA-9-450-s004.docx]

**Appendix S4**. **Duration of post-exposure period**

Statistical analysis

We analyzed effects of population type, density and the pesticide concentration on the duration of the post-exposure period using an ANOVA. In the model, population type, density and pesticide were included as fixed factors and population nested in the population type was included as a random factor. Containers were used as the unit of replication.

Results

The duration of the post-exposure period was shorter at the low density (ca. 20 days) than at the high density treatment (ca. 28 days) (Density, F_1, 67_ = 114.70, *P* < 0.001, Fig. S3). The duration of post-exposure period was ca. 3 days longer in larvae that were previously exposed to esfenvalerate (F_2, 67_ = 4.81, *P* = 0.011). The duration of the post-exposure period did not differ between edge and core populations (F_1, 2_ = 0.06, *P* = 0.83). There were no interactions between population type, density and esfenvalerate exposure on the post-exposure duration (all *P* > 0.19).


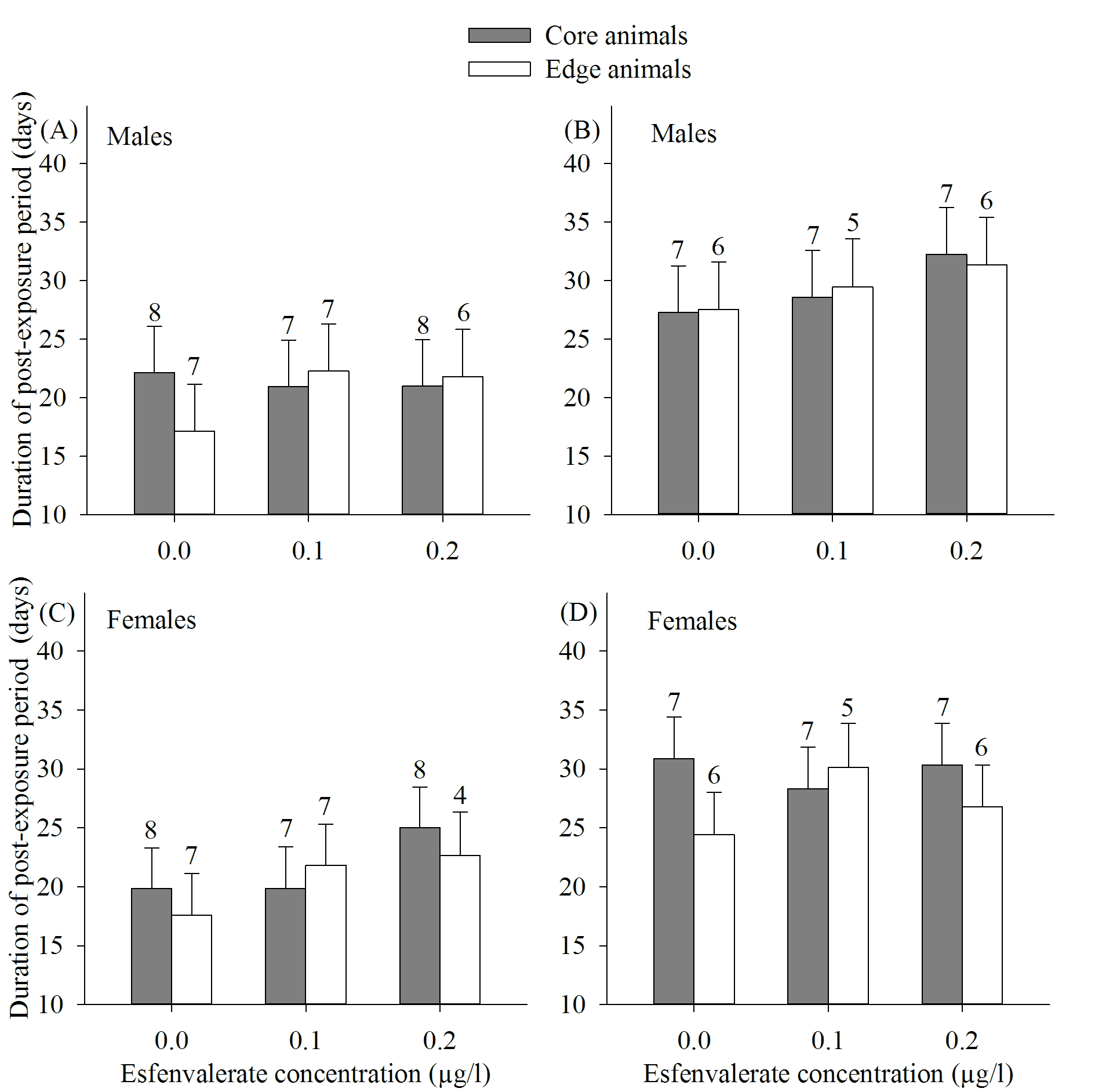


**Figure S3**. Duration of the post-exposure period of males (A, B) and females (C, D) of the damselfly *Coenagrion scitulum* as a function of esfenvalerate concentration, density and population type. Numbers above the bars represent the number of container replicates. Means are given with 1 SE.
