# Supplementary material for: Rapid evolution of increased vulnerability to an insecticide at the expansion front in a poleward‐moving damselfly
Source: Evol Appl. 2016 Jan 27;9(3):450–61. doi: 10.1111/eva.12347 (PMC4778112; doi:10.1111/eva.12347)
Supplement: Supplementary file 5 — Appendix S5. Mortality and growth rate of damselfly larvae during the exposure period. Figure S4. Mortality (A, B) and growth rate (C, D) of Coenagrion scitulum damselfly larvae during the exposure period as a function of esfenvalerate concentration, density and population type. [file EVA-9-450-s005.docx]

**Appendix S5: Mortality and growth rate of damselfly larvae during the exposure period**

**
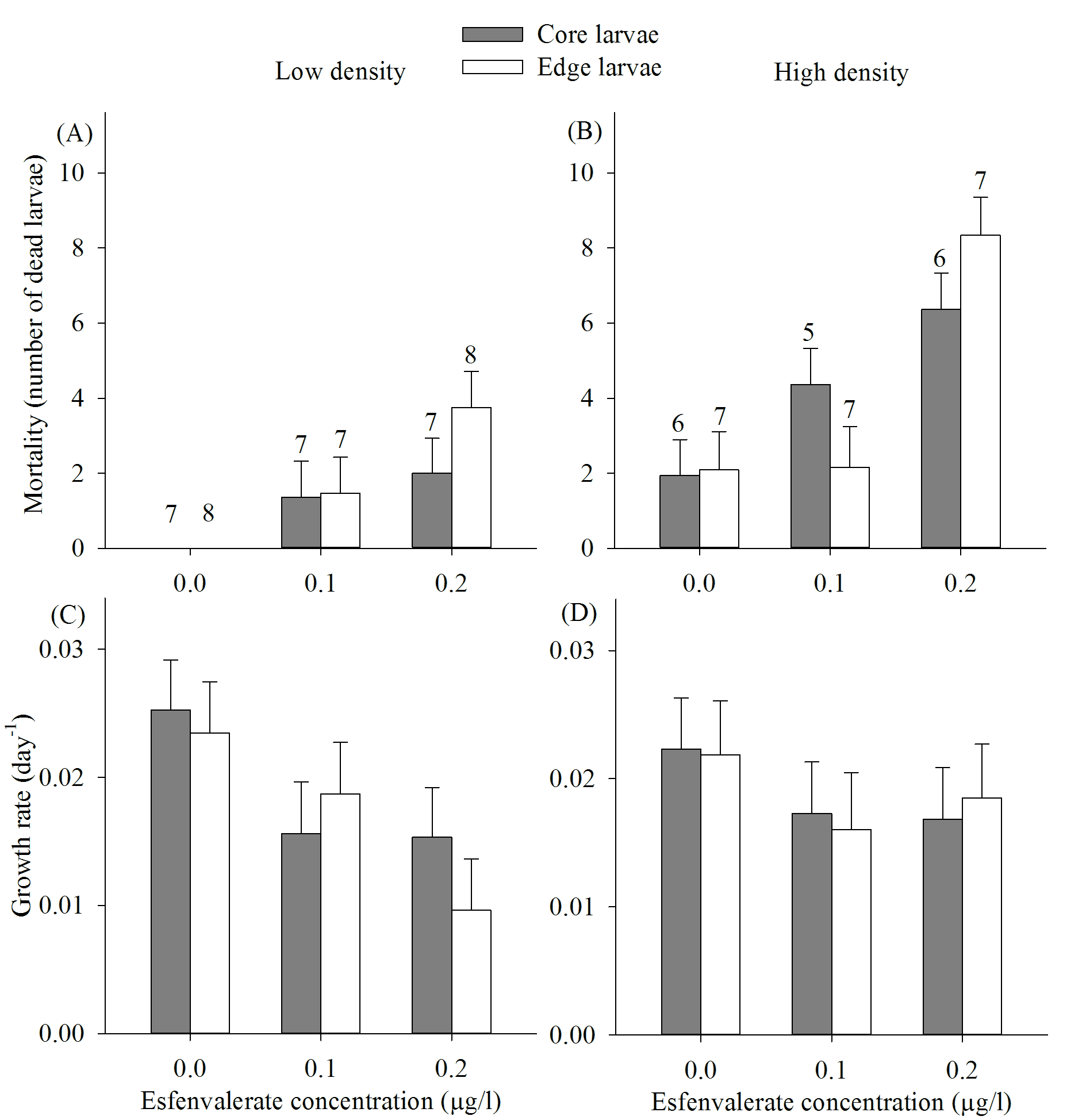
**

**Figure S4.** Mortality (A, B) and growth rate (C, D) of *Coenagrion scitulum* damselfly larvae during the exposure period as a function of esfenvalerate concentration, density and population type. Numbers above the bars represent the number of container replicates. Least-square means are given with 1 SE.
